# Characterization of the *Rbfox3*‐IRES‐iCre knock‐in mouse: Revealing gene recombination activity in neural and non‐neural peripheral tissues

**DOI:** 10.1096/fba.2024-00143

**Published:** 2025-02-13

**Authors:** Shiho Nishino, Misuzu Hashimoto, Swapna Paramanya Biswas, Natsuki Mikami, Yoshikazu Hasegawa, Hayate Suzuki, Woojin Kang, Seiya Mizuno, Kazuya Murata

**Affiliations:** ^1^ Laboratory Animal Resource Center in Transborder Medical Research Center, Institute of Medicine University of Tsukuba Tsukuba Ibaraki Japan; ^2^ Laboratory of Biological Chemistry, Faculty of Applied Biological Sciences Gifu University Gifu Japan; ^3^ Program in Human Biology, School of Integrative and Global Majors University of Tsukuba Tsukuba Japan; ^4^ Japan Society for the Promotion of Science Chiyoda‐ku Japan; ^5^ Center for One Medicine Innovative Translational Research (COMIT), Institute for Advanced Study Gifu University Gifu Japan

**Keywords:** bladder, Cre‐loxP, gene recombination, germline, heart, knock‐in mice, nervous system, NeuN, neuron, RBFOX3

## Abstract

In vivo cell type‐specific genetic recombination based on the Cre‐loxP system has contributed to the understanding of biological processes and diseases. Neuronal nuclei (NeuN)/RBFOX3 is a widely used mature neuron marker in developmental biology and neuroscience. Here, we generated *Rbfox3*‐improved Cre (iCre) knock‐in mouse model and investigated the effect of iCre knock‐in into the *Rbfox3* gene and Cre recombination activity in the central nervous system (CNS) and peripheral tissues. The knock‐in of internal ribosome entry site (IRES)‐iCre cassette into the *Rbfox3* 3′ UTR did not affect birth rate, growth, and brain weight. In the adult brain, iCre protein expression was confirmed, whereas RBFOX3 protein expression was partially reduced in the knock‐in mice. Cre recombination analysis using R26GRR fluorescent reporter strain revealed that *Rbfox3*‐driven iCre‐induced gene recombination in the CNS and heart during embryonic development. In the adult brain, gene recombination was observed in neurons, however, not in other glial cells. In the peripheral tissues, iCre activity was found in the sciatic nerve and in other peripheral tissues, including the heart, bladder, and testis. We validated gene recombination rate in the germline and found that 100% recombination occurred in male germ cells and approximately 50% in female germ cells. Concludingly, *Rbfox3*‐iCre mice induce genetic recombination in neurons within CNS as well as in some peripheral tissues and germ cells. In addition to establishing a novel Cre mouse line, the findings of this study offer valuable insights into the development and application of mouse tools that utilize the *Rbfox3* gene locus.

## Graphical abstract

This study developed an *Rbfox3*‐iCre knock‐in mouse model to explore Cre‐loxP‐based genetic recombination. Gene recombination was observed in the central nervous system and heart during embryonic development and in neurons, but not glial or NeuN‐negative neural cells, in the adult brain. Peripheral recombination occurred in the sciatic nerve, heart, bladder, and testis. This work establishes a new Cre mouse line and provides valuable insights for developing and applying genetic tools targeting the *Rbfox3* gene locus.

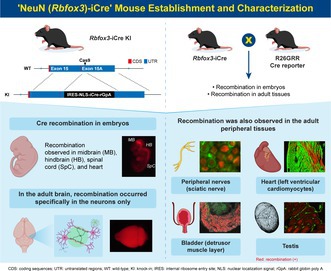

## 1 INTRODUCTION

Neuronal nuclei (NeuN) is a monoclonal antibody that specifically recognizes mature postmitotic neurons.^1^, ^2^ Anti‐NeuN antibodies have been extensively used in neuroscience research and developmental biology, especially for the visualization of neurons by immunostaining or the isolation of neuronal nuclei by fluorescence‐activated cell sorting.^3^, ^4^ In 2009, biochemical analysis using mass spectrometry revealed that NeuN was encoded by *Rbfox3* gene.^5^ RBFOX3 belongs to the RBFOX family of splicing regulators and is associated with the regulation of neuronal function.^6^ Deletion of *Rbfox3* gene has been shown to affect seizure susceptibility, anxiety‐related behavior, and cognitive abilities in mice.^7^, ^8^ These findings highlight that *Rbfox3* is an excellent marker for mature neurons and also plays a critical role as a gene involved in the regulation of neuronal functions.

Conditional knockout mouse models based on Cre‐loxP, a gene recombination system originally found in bacteria, have contributed to the understanding of how genes regulate cellular functions in the central nervous system (CNS).^9^ Many types of Cre driver mice that express Cre recombinase under tissue‐ or cell‐type‐specific promoters have been developed and are widely applied not only for gene targeting but also for optogenetics, cellular visualization, and lineage tracing.^10^ Currently, two knock‐in mouse models that harbor *Rbfox3*‐driven Cre or Dre, another site‐specific recombinase, have been developed. Both *Rbfox3* ^*em1(2A‐DreERT2‐WPRE‐pA)Smoc*^ ^11^ and *Rbfox3* ^*tm1(EGFP/Cre/ERT2)Wtsi*^ (EM:12292, The European Mouse Mutant Archive) mice were knocked in with tamoxifen‐inducible Cre/Dre at the endogenous *Rbfox3* locus, however, detailed information, such as the exact knock‐in site and Cre/Dre expression patterns in the tissue, is not yet available. Furthermore, tamoxifen‐inducible gene recombination system enables temporal control of genetic modification while presenting certain disadvantages, such as low gene knockout efficiency in the brain and the adverse effects of tamoxifen on pregnant mice and their embryos.^12^, ^13^, ^14^ Thus, it may be preferable to use a non‐inducible Cre driver depending on the stage or condition of the mouse model.

In this study, we generated an *Rbfox3*‐improved Cre (iCre) knock‐in mouse model and evaluated its characteristics. Heterozygous and homozygous knock‐in of the internal ribosome entry site (IRES)‐iCre cassette to the endogenous *Rbfox3* gene affected RBFOX3 protein expression in the brain, however, not body weight, brain weight, and neurogenesis in adult mice. iCre protein expression was detected in the brains of adult knock‐in mice. Breeding with Cre reporter mice revealed that iCre‐mediated gene recombination occurs in the CNS and heart during embryonic development. In the adult brain, recombination has been observed in neurons, however, not in oligodendrocytes, astrocytes, or microglia. The sciatic nerve, a part of the peripheral nervous system, also undergoes gene recombination via iCre. However, ectopic gene recombination has also been detected in some non‐neural cells, including cardiomyocytes, detrusor smooth muscle cells, and male germ cells. We also observed high frequencies of germline recombination in both male and female mice. This study established a novel Cre driver line for neuroscience and offers valuable insights into the development and application of mouse tools that utilize the *Rbfox3* gene locus.

## 2 MATERIALS AND METHODS

### 2.1 Animals

ICR and C57BL/6 mice were purchased from Jackson Laboratory (Yokohama, Japan). C57BL/6J‐*Rbfox3* ^*em1(IRES‐iCre)Murk*^ (*Rbfox3*‐iCre) mice were used in this study. The Cre driver line was deposited at the RIKEN BioResource Research Center. The depository number is RBRC12178. R26GRR mice (RBRC04874), a reporter line for Cre activity, have been described previously.^15^ Animals were housed in plastic cages under specific pathogen‐free conditions in a room maintained at 23.5°C ± 2.5°C and 52.5% ± 12.5% relative humidity under a 14:10‐h light: dark cycle. Mice had free access to commercial chow (MF; Oriental Yeast, Tokyo, Japan) and filtered water. Breeding and experiments were performed in accordance with the Regulations for Animal Experiments of the University of Tsukuba and the Fundamental Guidelines for Proper Conduct of Animal Experiments and Related Activities in Academic Research Institutions under the jurisdiction of the Ministry of Education, Culture, Sports, Science, and Technology of Japan. The study was approved by the Institutional Animal Experiment Committee of the University of Tsukuba (Approval number: 23‐067).

### 2.2 Establishment of Rbfox3‐iCre mice

**FIGURE 1:**
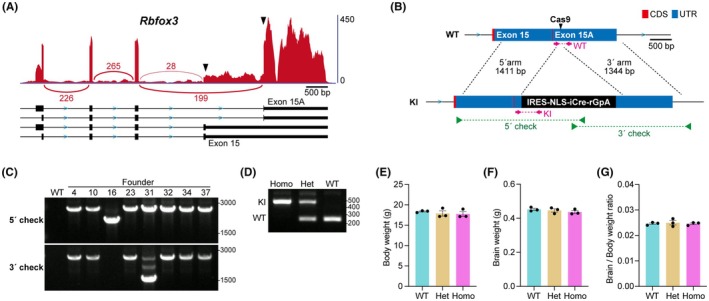
Generation of *Rbfox3*‐IRES‐iCre mice. (A) Sashimi plot image of *Rbfox3* transcripts around the last exon in neurons. The position of stop codon is indicated by black arrowheads. (B) The knock‐in strategy of IRES‐NLS‐iCre‐rGpA cassette. Cleavage site by Cas9 is indicated by black arrowhead in the wild‐type (WT) allele. The position of primer is indicated by green arrowheads (for founder screening) and pink arrows (for routine genotyping) in the WT and knock‐in (KI) allele. (C) Founder screening by genomic PCR using 5′ and 3′ check primer sets. Product lengths of each PCR are as follows: 5′ check, 2713 bp; 3′ check, 2685 bp. (D) Representative image of routine genotyping. Product lengths of each PCR are as follows: WT allele, 251 bp; KI allele, 487 bp. (E–G) Body weight, brain weight, and brain per body weight ratio of 7–8‐week‐old female *Rbfox3*‐iCre mice. All data are presented as mean ± SEM. Individual values are shown as dots in the graph. One‐way ANOVA test revealed that no significant differences were present in any parameter between WT, heterozygote (Het), and homozygote (Homo).

*Rbfox3*‐iCre mice were generated via zygote genome editing using the CRISPR/Cas9 system. An IRES‐nuclear localization signal (NLS)‐iCre‐rabbit globin poly A signal (rGpA) sequence was inserted into 3′ UTR of the *Rbfox3* gene by homologous recombination (Figure 1B). The synthetic crRNA, including the target sequence (5′‐ATGCGGGTGTACGCCAGACG‐3′), and tracrRNA was obtained from Integrated DNA Technologies (Skokie, IL, USA). To construct the donor vector, 1411 bp upstream and 1344 bp downstream of the Cas9 cleavage site in *Rbfox3* gene were cloned at both ends of the IRES‐NLS‐iCre‐rGpA cassette (Figure 1B). The CRISPR/Cas9 ribonucleoprotein complex and donor DNA were microinjected into the zygotes of C57BL/6J mice (Jackson Laboratory Japan, Kanagawa, Japan) according to our previous report.^16^ Subsequently, microinjected zygotes were transferred into the oviducts of pseudopregnant ICR females (Jackson Laboratory Japan, Kanagawa, Japan), and newborns were obtained.

### 2.3 Genotyping

A small piece of ear harvested from 10‐ to 12‐day‐old mice or tail harvested from embryos was incubated with lysis buffer (0.5 M Tris‐HCl, 0.1 M EDTA, 0.1 M NaCl, 1% SDS) containing 0.3 mg/mL of proteinase K (Cat. #25530049, Thermo Fisher Scientific, Waltham, MA, USA) in a thermomixer at 60°C at 850 rpm for 3 h. The samples were then cooled to room temperature and added to the RNase solution. After the incubation at 37°C for 30 min, proteins were removed from samples by adding ammonium acetate and centrifugation at 14,000 rpm for 10 min. Genomic DNA was precipitated by adding equal volume of isopropanol and centrifugation at 4°C at 14,000 rpm for 10 min. After washing the pellet with 70% ethanol, the DNA was dissolved in 10 mM Tris‐HCl and subjected to standard PCR using QuickTaq HS DyeMix (Cat. #DTM‐101, TOYOBO, Osaka, Japan) according to the manufacturer's instructions. The PCR products were separated by 0.7% or 1.5% agarose gel electrophoresis. 5′‐arm check‐F (5′‐CCCCTCACAGCATTCACACTAC‐3′), 5′‐arm check‐R (5′‐ATCAGCATTCTCCCACCATC‐3′), 3′‐arm check‐F (5′‐GTCCCTGGTGATGAGGAGAA‐3′), and 3′‐arm check‐R (5′‐ACAGTCTCCCCATTACCTCCTG‐3′) were used for detecting the regions outside of homology arm to insert in founder mice. *Rbfox3*‐iCre‐F (5′‐CAAGCAACAACAGCAACAACAA‐3′), *Rbfox3*‐iCre‐R1 (5′‐AGCAGGGTGGGAAAGGAAGA‐3′), and *Rbfox3*‐iCre‐R2 (5′‐ATACGCTTGAGGAGAGCCATTT‐3′) were used for routine genotyping of *Rbfox3*‐iCre mice. The genotype of R26‐GRR mice was determined using the following primers: ROSA26‐F (5′‐ACTTGCTCTCCCAAAGTCGC‐3′), ROSA26‐R (5′‐GGGAAGTCTTGTCCCTCCAAT‐3′), and CAG‐R (5′‐GGCGTACTTGGCATATGATACA‐3′).

### 2.4 Sashimi plot analysis

RNA‐seq data were downloaded from the National Center for Biotechnology Information (NCBI) Gene Expression Omnibus (GEO, accession #GSE52564).^17^ The data were analyzed using the CLC Genomics Workbench software (v23.0.1, Qiagen). Neuronal transcriptome reads were mapped to the mouse reference genome (GRCm38) using an RNA‐sequencing analysis tool. To visualize splicing events, BAM files were exported from the CLC Genomics Workbench, sorted, and imported into the Integrative Genomics Viewer (IGV), and Sashimi plot images were generated.

### 2.5 Western blotting

Brains were harvested from 7‐ to 8‐week‐old female mice and divided into left and right sides. The left brain was minced in RIPA buffer (Cat. #16488‐34, Nacalai Tesque, Kyoto, Japan), and homogenized using an ultrasonic homogenizer (cat. #LUH150, Yamato Scientific, Tokyo, Japan). After centrifugation at 4°C at 14,000 rpm for 20 min, the supernatant was subjected to protein concentration measurement using DC protein assay kit (Cat. #5000111, Bio‐Rad Laboratories, Hercules, CA, USA). The supernatants were mixed with 6x sodium dodecyl sulfate (SDS) sample buffer (Cat. #09499‐14, Nacalai Tesque), boiled for 3 min, and subjected to Western blotting. Western blotting was performed as previously described.^18^ The following antibodies were used: anti‐NeuN/RBFOX3 (1:1000; Cat. #94403; Cell Signaling Technology, Danvers, MA, USA), and anti‐Cre recombinase (1:200; cat. #15036, Cell Signaling Technology), anti‐β3‐Tubulin (1:1000, Cat. #5568, Cell Signaling Technology), anti‐glyceraldehyde 3‐phopshate dehydrogenase (GAPDH) (1:5000, #60004‐1‐IG, Proteintech, Rosemont, IL, USA), anti‐Neurofilament‐H (1:2000, Cat. #30564, Cell Signaling Technology), anti‐Neurofilament‐L (1:500, Cat. #2835, Cell Signaling Technology), and anti‐VAMP1 (1:1000, Cat. #13151, Cell Signaling Technology). The signal intensity of the bands was measured using ImageJ software.

### 2.6 RNA experiments

The right brains of 7–8‐week‐old female mice were harvested and homogenized in ISOGEN II (Cat. #311‐07361, Nippon Gene, Tokyo, Japan) using a bead‐based homogenizer (Micro Smash, Cat. #MS100, Tomy, Tokyo, Japan) at 4,000 rpm for 20 s, repeated three times. After centrifugation at room temperature at 14,000 rpm for 5 min, the supernatants were transferred to new tubes, mixed with ultrapure water (Cat. #10977015, Thermo Fisher Scientific), vortexed for 30 s, and incubated for 15 min. The mixture was then centrifuged at room temperature at 14,000 rpm for 15 min, and the supernatants were transferred to new tubes and mixed with isopropanol to precipitate RNA. The RNA pellets were washed with 70% ethanol, air‐dried, and dissolved in ultrapure water. Reverse transcription was performed using the ReverTra Ace qPCR RT master mix with gDNA remover (Cat. #FSQ‐301, TOYOBO) following the manufacturer's protocol. Quantitative PCR was carried out using GeneAce SYBR qPCR Mix α (Cat. #319‐07703, Nippon Gene) on a CFX Connect Real‐Time PCR Detection System (BioRad). Gene expression levels of *Rbfox3* and iCre were normalized using *Hprt1* gene expression. The primers used for quantitative PCR were as follows: q‐*Rbfox3*‐F (5′‐AGCAGCAGCCCAAACGACTA‐3′), q‐*Rbfox3*‐R (5′‐TTGGAGCCCCGCTCGTTAAA‐3′), q‐iCre‐F (5′‐CCTGGCTGTGAAGACCATCC‐3′), q‐iCre‐R (5′‐CCCAGCATCCACATTCTCCT‐3′), q‐*Hprt1*‐F (5′‐CTGGTTAAGCAGTACAGCCCC‐3′), and q‐*Hprt1*‐R (5′‐TCAAATCCAACAAAGTCTGGCCT‐3′).

For gene expression analysis at embryonic day 10.5, head and heart tissue were harvested from wild‐type embryos. Total RNA was extracted using the NucleoSpin RNA Plus XS (Cat. # U0990B, Takara, Shiga, Japan) and reverse‐transcribed using the ReverTra Ace qPCR RT master mix (Cat. #FSQ‐201, TOYOBO) according to the manufacturer's instructions. RT‐PCR was performed with the AmpliTaq Gold 360 Master Mix (Cat. # 4398881, Thermo Fisher Scientific). The PCR products were resolved by electrophoresis on a 1.5% agarose gel containing ethidium bromide. Gel images were captured using the iBright CL1500 imaging system (Thermo Fisher Scientific). The primers used for RT‐PCR were as follows: RT‐*Rbfox3*‐F (5′‐ACACAGCCCATTGCTGGGAC‐3′), RT‐*Rbfox3*‐R (5′‐CAGCCATTGGCATATGGGTTCC‐3′), RT‐*Sox2*‐F (5′‐GGAGGAGAGCGCCTGTTTTT‐3′), RT‐*Sox2*‐R (5′‐CTGGCGGAGAATAGTTGGGG‐3′), RT‐*Myh6*‐F (5′‐CAAGCTCACTTGAAGGACACC‐3′), RT‐*Myh6*‐R (5′‐CACGATGGCGATGTTCTC‐3′), RT‐*Uba52*‐F (5′‐CGCTGTCCTCTTTCTCTTCAACG‐3′), and RT‐*Uba52*‐R (5′‐TACTTCTGGGCAAGCTGACGA‐3′).

### 2.7 Analysis of Cre recombination

Heterozygous *Rbfox3*‐iCre mice were crossed with homozygous R26GRR mice. At embryonic day 10.5 and 12.5 (E10.5 and E12.5), embryos were isolated from their mother and observed using the Leica M165 FC fluorescent stereo microscope (Leica Microsystems, Wetzlar, Germany). To observe adult tissues, 8‐week‐old mice were anesthetized and perfused with cold PBS (−) and 4% paraformaldehyde (Cat. #163‐20145, Fujifilm Wako Pure Chemical Industries Ltd., Osaka, Japan). Tissues were harvested and observed under a fluorescent stereomicroscope (Leica Microsystems). The tissues were further fixed with 4% paraformaldehyde for 24 h at 4°C, immersed 30% sucrose at 4°C with gentle shaking overnight, and embedded in Tissue‐Tek O.C.T. Compound (Cat. #45833, Sakura Finetek, Tokyo, Japan). The sciatic nerve, heart, bladder, and testes were dissected into 10 μm sections and air‐dried for 2 h. After washing with PBS (−), the coverslips were mounted using FluoSave Reagent (Cat. #345789, Merck, Darmstadt, Germany). Fluorescence images were obtained using an EVOS M5000 imaging system (Thermo Fisher Scientific).

### 2.8 Immunohistochemistry

The brains were sectioned into 10 μm sections and air‐dried. After washing three times with PBS (−), the sections were incubated with 10% normal goat serum for 60 min and incubated with primary antibodies at 4°C overnight. Sections were washed thrice with PBS (−) and incubated with a secondary antibody at room temperature for 60 min. Nuclei were visualized by staining with Hoechst33342 (Cat. #346‐07951, Fujifilm Wako Pure Chemical). After washing with PBS (−), coverslips were mounted using Fluoromount‐G (Cat. # 0100‐01, SouthernBiotech, Birmingham, AL, USA). The following antibodies were used: anti‐DCX (1:500, Cat. #4604, Cell Signaling Technology), anti‐NeuN/RBFOX3 (1:100; Cat. #24307, Cell Signaling Technology), anti‐β3‐Tubulin (1:200, Cat. #5568; Cell Signaling Technology), anti‐calbindin (1:500; Cat. #ab108404; Abcam, Cambridge, UK), anti‐Calretinin (1:100, Cat. #92635, Cell Signaling Technology), anti‐MBP (1:100; Cat. #MAB386; Merck), anti‐glial fibrillary acidic protein (GFAP; 1:200; cat. #13‐0300, Thermo Fisher Scientific), anti‐IBA1 (1:500; Cat. #019‐19741, Fujifilm Wako Pure Chemical), Cy5 AffiniPure donkey anti‐rabbit IgG (H + L) (1:500, Cat. #711‐175‐152; Jackson ImmunoResearch, West Grove, PA, USA), AlexaFluor488 AffiniPure donkey anti‐Rabbit IgG (H + L) (1:500, Cat. #711‐545‐152, Jackson ImmunoResearch), and Alexa Fluor 647‐conjugated goat anti‐rat IgG (H + L) cross‐adsorbed secondary antibodies (1:1000; Cat. #A‐21247, Thermo Fisher Scientific). Fluorescent images were obtained using the BZ‐X810 imaging system (Keyence, Osaka, Japan). DCX positive cells were quantified in the hippocampal dentate gyrus of adult wild‐type and heterozygous *Rbfox3*‐iCre mice. For quantification of images, three mice per each genotype were used, and dentate gyrus areas in the coronal brain sections were applied to manual cell counting with the ImageJ software. For each mouse, up to six areas from both the left and right dentate gyrus were used. In total, 34 areas were measured for wild‐type mice and 31 areas for heterozygous mice.

### 2.9 Analysis of germline recombination

*Rbfox3*‐iCre::R26GRR mice were crossed with C57BL/6 mice. Five male and four female *Rbfox3*‐iCre::R26GRR mice were used in this analysis. Embryos were isolated from their mothers on embryonic days 18 or 19 and observed using a Leica M165 FC fluorescent stereomicroscope (Leica Microsystems). Tail genomes of the embryos were genotyped. A total of 35 and 25 pups were analyzed for male and female *Rbfox3*‐iCre::R26GRR mice, respectively.

### 2.10 Statistical analysis

Statistical analyses were performed using the GraphPad Prism 8 software (GraphPad Prism Software). Data were analyzed using unpaired *t*‐test, one‐way ANOVA, and Welch's *t*‐test. Differences were considered statistically significant at *p* < 0.05.

## 3 RESULTS

### 3.1 Generation of Rbfox3‐iCre knock‐in mice

The bicistronic expression of an exogenous gene under the control of an endogenous promoter using 2A or IRES is widely applied in Cre driver mouse models.^19^ To determine the knock‐in site of the bicistronic Cre expression cassette, we analyzed the exon usage pattern of *Rbfox3* mRNA in neurons. Mouse *Rbfox3* gene has alternative 3′ splice sites in its last exon (Figure 1A). The use of exon 15 or 15A produces different isoforms of RBFOX3. Exon 15 encodes the hydrophobic proline‐tyrosine nuclear localization sequence (hPY‐NLS), whereas exon 15A has only a stop codon, resulting in different cellular localizations between RBFOX3 isoforms.^20^ We observed that the use of exon 15A in neurons was six to seven times higher than that of exon 15 (Figure 1A). Therefore, exon 15A was selected as the Cre knock‐in site (Figure 1B). A donor vector, including the homology arms and IRES‐NLS‐iCre‐rGpA cassette, was constructed and used to generation *Rbfox3*‐iCre mice. We obtained six founder mice with a knock‐in allele (Figure 1C) and established an *Rbfox3*‐iCre mouse line using one of these founder mice. Homozygous knock‐in mice were obtained by intercrossing heterozygous mice (Figure 1D). Heterozygous and homozygous knock‐in mice grew normally and had body and brain weights similar to those of wild‐type mice (Figure 1E–G).

### 3.2 Endogenous RBFOX3 and iCre expression in the brain

**FIGURE 2:**
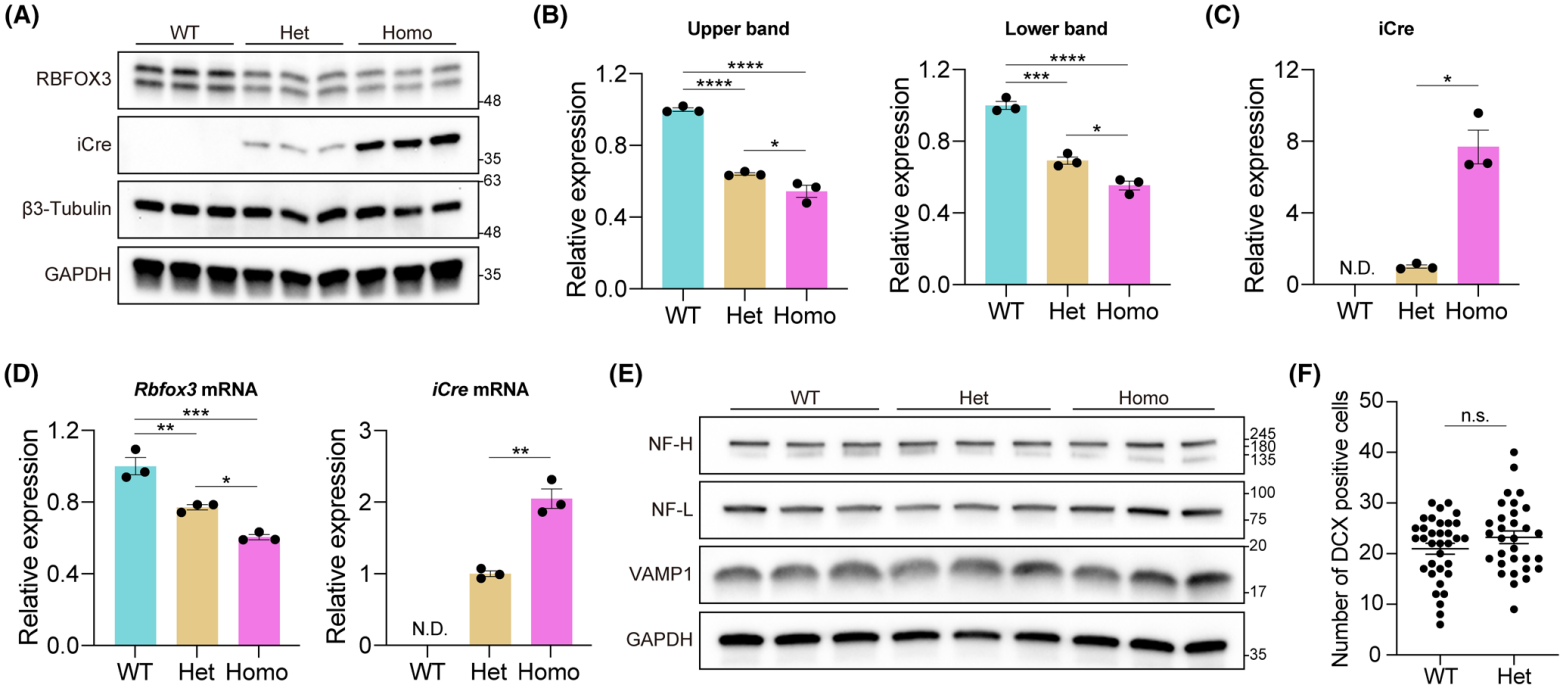
RBFOX3 and iCre expression in the brain of *Rbfox3*‐iCre mice. (A) Western blotting analysis of RBFOX3, iCre, β3‐Tubulin, and GAPDH in the brain of wild‐type (WT), heterozygote (Het), and homozygote (Homo). β3‐Tubulin and GAPDH were used as the internal control. Molecular mass is indicated alongside the blots. (B) Quantification of RBFOX3 protein isoforms expression. All data are presented as mean ± SEM. Individual values are shown as dots in the graph. *p < 0.05; ***p < 0.001; ****p < 0.0001, one‐way ANOVA followed by Tukey's multiple comparisons test. (C) Quantification of iCre protein expression. All data are presented as mean ± SEM. Individual values are shown as dots in the graph. *p < 0.05, Welch's *t*‐test. (D) Relative mRNA expression of *Rbfox3* and iCre gene. All data are presented as mean ± SEM. Individual values are shown as dots in the graph. *p < 0.05; **p < 0.01; ***p < 0.001, one‐way ANOVA followed by Tukey's multiple comparisons test in left panel. **p < 0.01, unpaired *t*‐test in the right panel. (E) Western blotting analysis of neurofilament heavy chain (NF‐H), neurofilament light chain (NF‐L), and vesicle‐associated membrane protein 1 (VAMP1) in the brain of WT, Het, and Homo. GAPDH was used as the internal control. Molecular mass is indicated alongside the blots. (F) Evaluation of adult neurogenesis in the hippocampus. Doublecortin (DCX)‐positive cells, an important marker of neurogenesis, were counted. All data are presented as mean ± SEM. Individual values are shown as dots in the graph.

3′ UTR of mRNA contains some regulatory elements for the mRNA stability and translation.^21^ Previous studies showed that knock‐in of IRES‐Cre or P2A‐Flpo cassette into the 3′ UTR of the *Slc6a3* (DAT) gene results in reduced endogenous DAT protein expression.^22^, ^23^ To investigate the endogenous RBFOX3 and iCre expression levels, Western blotting and qPCR analyses were performed using the brain tissue. iCre protein expression was successfully detected in heterozygous and homozygous knock‐in mice (Figure 2A) and was seven–eight times higher in homozygous knock‐in mice than that in heterozygotes (Figure 2C). On the contrary, the iCre mRNA levels were twice as high in homozygous mice compared to heterozygous mice (Figure 2D), suggesting that the marked increase in iCre protein levels may be attributed to factors such as protein stability.

We found that RBFOX3 protein expression levels were significantly reduced in knock‐in mice compared to those in wild‐type mice (Figure 2A,B). However, RBFOX3 expression in homozygous knock‐in mice remained approximately 50% of that in wild‐type mice (Figure 2B). Furthermore, qPCR analysis revealed that *Rbfox3* mRNA expression was reduced in the brains of knock‐in mice (Figure 2D), with the degree of reduction comparable to that observed at the protein level, indicating that the knock‐in of IRES‐iCre cassette may have impaired transcription or mRNA stability of *Rbfox3*.

Previous studies have demonstrated that the loss of *Rbfox3* results in reduced brain weight, reduced expression of neurofilament proteins, and impaired adult neurogenesis in the hippocampus.^7^, ^8^ Another study reported that selective deletion of *Rbfox3* in GABAergic neurons leads to the downregulation of vesicle‐associated membrane protein 1 (VAMP1) expression, causing a seizure phenotype.^24^ However, Western blot analysis clearly shows that the protein expression levels of Neurofilament‐H, Neurofilament‐L, and VAMP1 were not affected in *Rbfox3*‐iCre mice (Figure 2E). Furthermore, adult neurogenesis was not impaired in *Rbfox3*‐iCre mice (Figure 2F). These findings indicate that the *Rbfox3*‐iCre mouse is not a loss‐of‐function model, such as the *Rbfox3*‐KO mouse.

### 3.3 Cre recombination activity in the embryos

**FIGURE 3:**
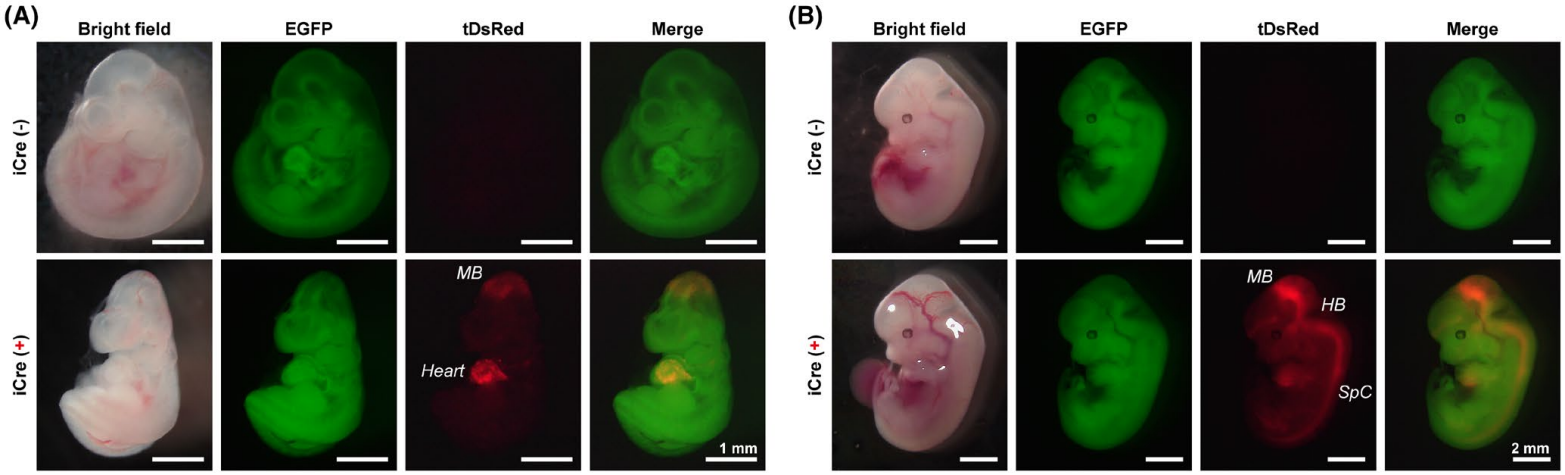
Cre recombination in the *Rbfox3*‐iCre::R26GRR embryos. (A and B) Representative images of R26GRR embryos with or without *Rbfox3*‐iCre allele at embryonic day 10.5 (E10.5) and E12.5. Scale bars are shown in each panel. The position of midbrain (MB), heart, hindbrain (HB), and spinal cord (SpC) are indicated in figures.

To investigate whether iCre expression was sufficient to induce gene recombination and where recombination occurs, *Rbfox3*‐iCre mice were crossed with R26GRR reporter mice, which are fluorescent Cre reporters. R26GRR mice lacking Cre express enhanced green fluorescent protein (EGFP), whereas tdsRed expression is induced instead of EGFP by Cre recombination.^15^ A previous immunohistochemical study using an anti‐NeuN antibody demonstrated immunoreactivity in the midbrain at E10.5,^1^ indicating that *Rbfox3* gene begins to be expressed during early embryonic brain development. Consistent with this, tDsRed signals were detected in the midbrain of *Rbfox3*‐iCre::R26GRR embryos at E10.5 (Figure 3A, lower panels). At E12.5, tDsRed signals were detected not only in the midbrain but also in the hindbrain and spinal cord (Figure 3B). In R26GRR mice without *Rbfox3*‐iCre allele, tDsRed signals were not observed throughout the body (Figure 3A,B, upper panels). On the contrary, the heart also showed tDsRed signals in *Rbfox3*‐iCre::R26GRR embryos at E10.5 (Figure 3A, lower panels). Gene expression analysis revealed that the *Rbfox3* gene was expressed in the embryonic heart, even though its expression was lower compared to that in the brain (Figure S1). These data demonstrate that bicistronically expressing iCre from *Rbfox3*‐IRES‐iCre allele was able induced gene recombination in the embryonic CNS and heart, reflecting the endogenous expression pattern of *Rbfox3*.

### 3.4 Cre recombination in the adult tissues

**FIGURE 4:**
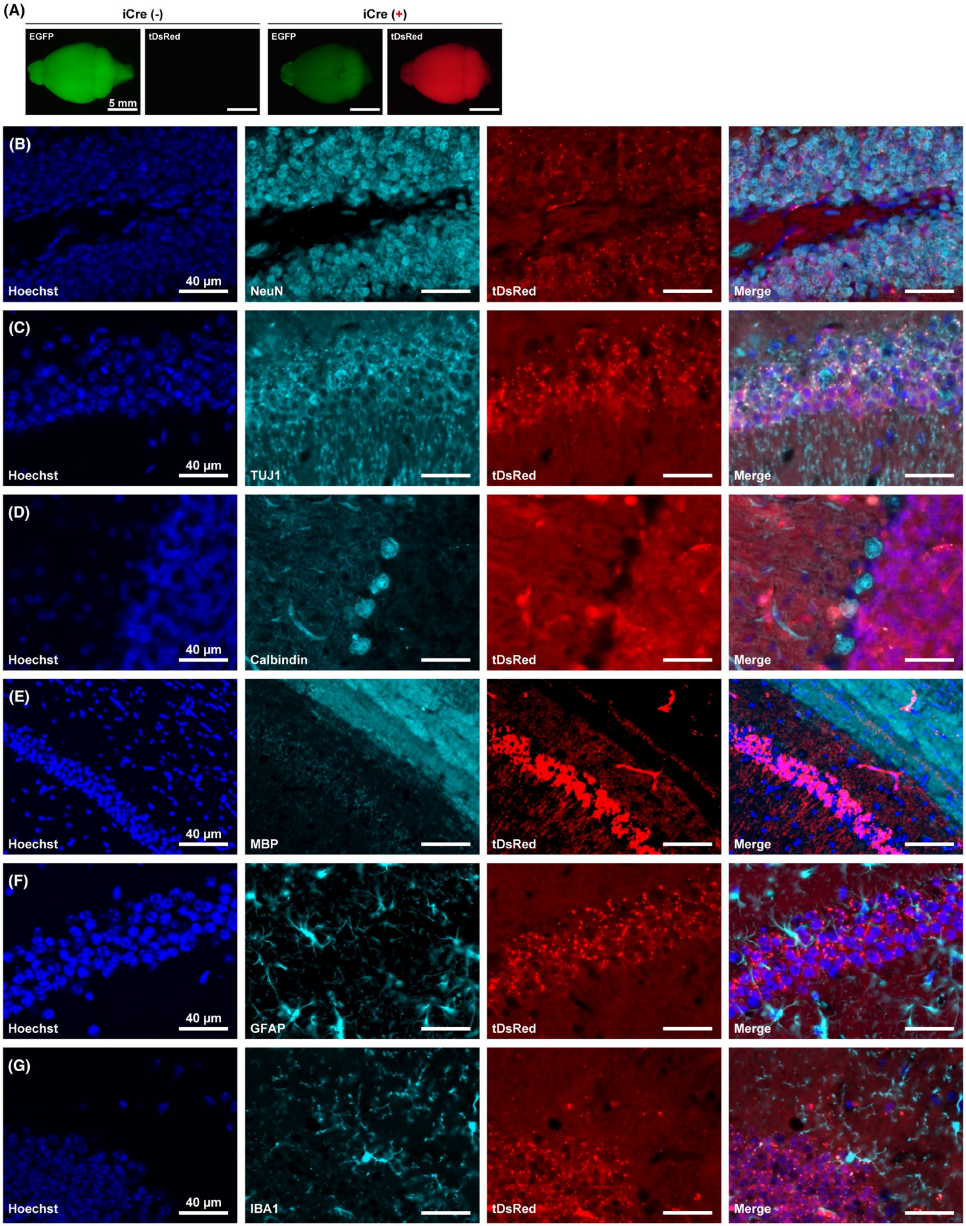
Cre recombination in the adult brain of *Rbfox3*‐iCre::R26GRR mice. (A) Representative fluorescent images of the brain harvested from R26GRR mice with or without *Rbfox3*‐iCre allele. Scale bars are shown in each panel. (B–G) Representative images of immunostaining of markers for various cell type in the brain section of *Rbfox3*‐iCre::R26GRR mice. Scale bars are shown in each panel. (B and C) NeuN and TUJ1 indicate mature neurons. (D) Calbindin indicates Purkinje cells. (E) Myelin basic protein (MBP) indicates mature oligodendrocytes. (F) Glial fibrillary acidic protein (GFAP) indicates astrocytes. (G) Ionized calcium‐binding adapter molecule 1 (IBA1) indicates microglia.

Further, we investigated the cell‐type specificity of Cre recombination in the brains of adult *Rbfox3*‐iCre::R26GRR mice. In the R26GRR mice, only EGFP signals were detected in the brain (Figure 4A, left panel). Conversely, strong tDsRed signals and reduced EGFP signals were observed throughout the brain of *Rbfox3*‐iCre::R26GRR mice (Figure 4A, right). Immunohistochemical analysis revealed that the distribution of tDsRed signals largely overlapped with that of the mature neuron markers NeuN/RBFOX3 and TUJ1 in the hippocampus (Figure 4B,C). In the cerebellum, tDsRed signals were not detected in Purkinje cells labeled with the calbindin antibody (Figure 4D). This result is consistent with a previous report showing that Purkinje cells do not react with the anti‐NeuN/RBFOX3 antibody.^1^, ^25^ Moreover, we found that gene recombination was absent in olfactory bulb mitral cells, which were also shown to lack immunoreactivity against anti‐NeuN/RBFOX3 antibody^1^ (Figure S2). The distribution of other glial cell markers, MBP (as mature oligodendrocyte marker), GFAP (astrocyte marker), and IBA1 (microglia marker), did not correspond to the tDsRed signals (Figure 4E–G). These data demonstrate that gene recombination occurs in mature neurons, however, not in NeuN (*Rbfox3*)‐negative neural cells, such as Purkinje cells, mitral cells, and other glial cells in the brains of *Rbfox3*‐iCre::R26GRR mice.

**FIGURE 5:**
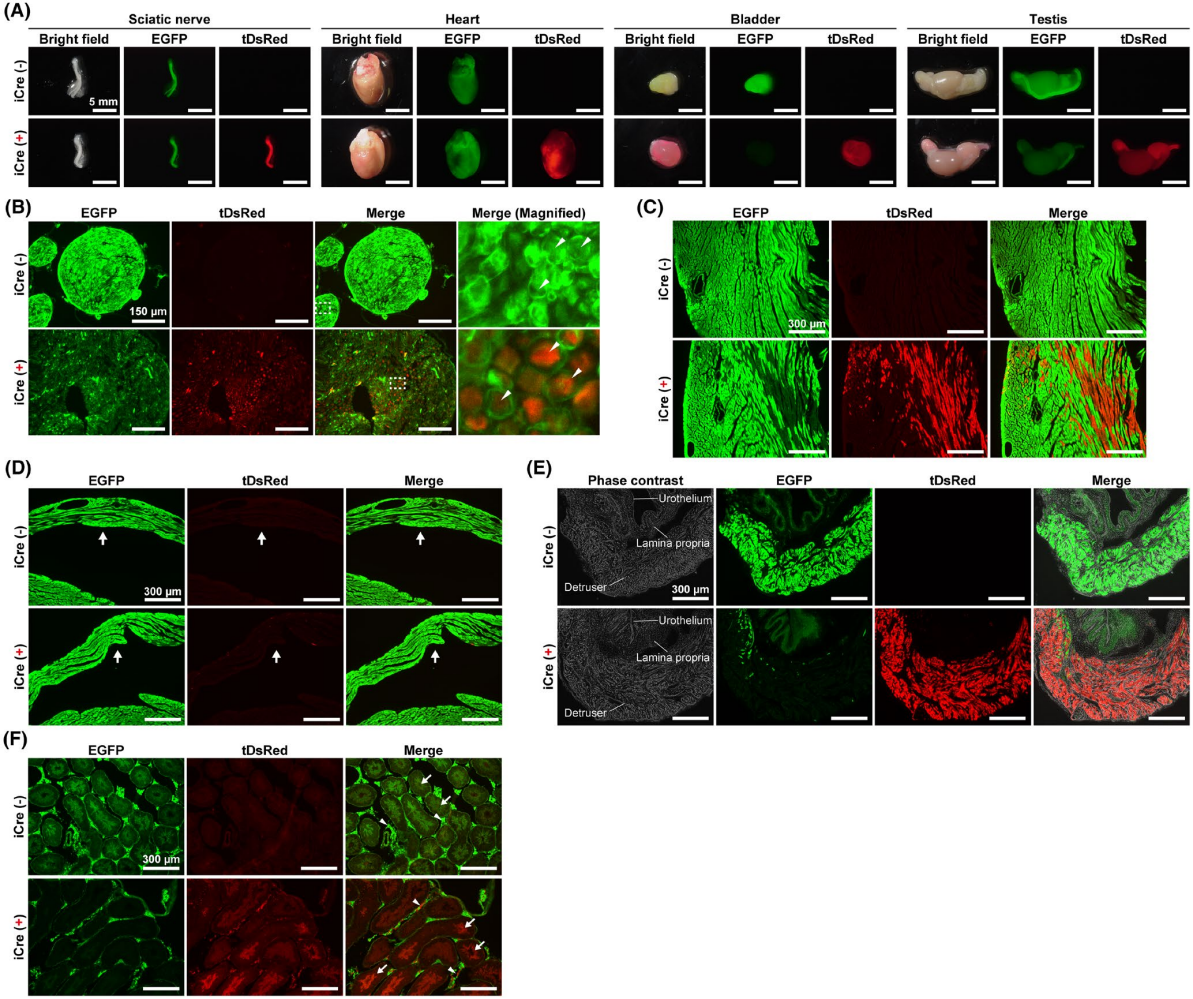
Cre recombination in the adult peripheral tissues of *Rbfox3*‐iCre::R26GRR mice. (A) Representative fluorescent images of the sciatic nerve, heart, bladder, and testis harvested from R26GRR mice with or without *Rbfox3*‐iCre allele. Scale bars are shown in each panel. (B–F) Representative fluorescent images of the tissue section. Scale bars are shown in each panel. (B) The sciatic nerve. The field of magnified images is indicated by white dotted square in merge images. Arrowheads indicate axons. (C) The left ventricle. (D) The right ventricle. Arrows indicate right ventricular walls. (E) The bladder. (F) The testis. Arrows indicate seminiferous tubules. Arrowheads indicate interstitial cells.

As ectopic Cre recombination was observed in the embryonic heart (Figure 3A), we further investigated Cre recombination activity in the peripheral tissues of adult mice. Stereo microscopic observations of fluorescence in *Rbfox3*‐iCre::R26GRR mice revealed that some tissues, such as the sciatic nerve, heart, bladder, and testes, had strong tDsRed signals (Figure 5A). The sciatic nerve, which is the largest peripheral nerve, is a bundle of nerve fibers myelinated by Schwann cells.^26^ In the cross‐section of the sciatic nerve, islet‐like tDsRed signals surrounded by EGFP signals were detected in *Rbfox3*‐iCre::R26GRR mice (Figure 5B), suggesting Cre recombination in peripheral neural cells. In the cardiac sections, tDsRed‐positive cardiomyocytes were abundant in the left ventricular wall (Figure 5C), whereas tDsRed signals were scarcely detected in the right ventricle (Figure 5D). The bladder consists of the urothelium, lamina propria, detrusor, and serosa.^27^ Cre recombination was found in most areas of the detrusor, however, not in the urothelium (Figure 5E). Although neither EGFP nor tDsRed signals were observed in the lamina propria or serosa, this may have been due to insufficient CAG promoter activity. In the testis, almost all seminiferous tubes contained a large number of tDsRed‐positive cells in *Rbfox3*‐iCre::R26GRR mice (Figure 5F, arrows). In addition, approximately 50% of the interstitial cells showed tDsRed signals (Figure 5F, arrowheads). These results indicate that Cre recombination was induced in *Rbfox3*‐iCre::R26GRR mice, not only in neurons but also in various cell types, including cardiomyocytes, detrusor smooth muscle cells, and male germ cells.

### 3.5 Germline Cre recombination rate in Rbfox3‐iCre::R26GRR mice

**FIGURE 6:**
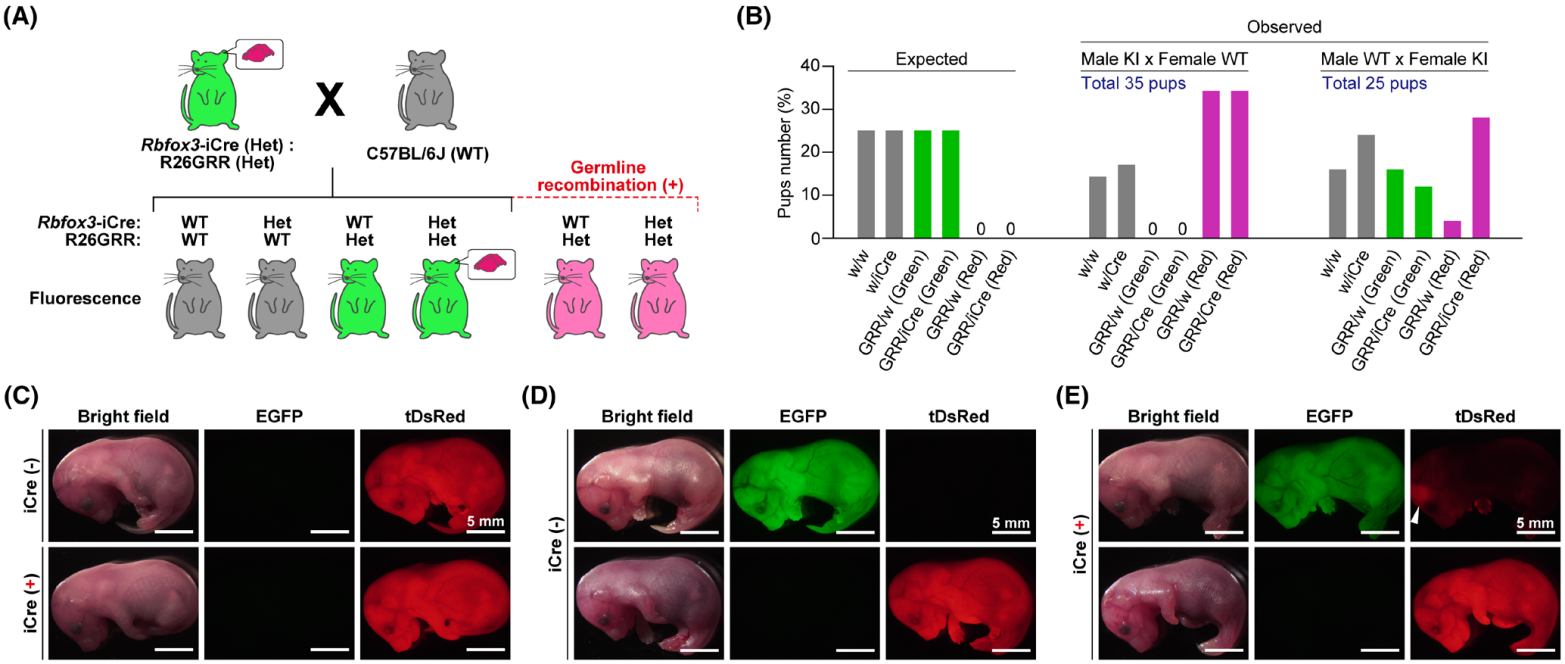
Germline recombination rate in *Rbfox3*‐iCre::R26GRR mice. (A) Schematic of germline recombination analysis. Heterozygous (Het) *Rbfox3*‐iCre::R26GRR mice were crossed with C57BL/6J (WT) mice. (B) Expected and observed number of pups. Genotypes and dominant fluorescent color of pups are shown in X axis. KI means *Rbfox3*‐iCre::R26GRR mice. (C–E) Representative fluorescent images of R26GRR pups with or without *Rbfox3*‐iCre allele. Scale bars are shown in each panel. (C) Pups obtained by crossing male KI with female WT mice. (D and E) Pups obtained by crossing male WT with female KI mice. An arrowhead indicates tDsRed fluorescence from the brain.

Finally, we investigated the Cre recombination rate in both male and female germ cells by crossing *Rbfox3*‐iCre::R26GRR mice with wild‐type mice (Figure 6A). It was expected that no fluorescent mice or green fluorescent mice could be obtained using this crossing strategy without germline recombination (Figure 6B). If Cre recombination occurred in germ cells, red‐fluorescent mice were obtained (Figure 6A). When male *Rbfox3*‐iCre::R26GRR mice were crossed with female wild‐type mice, all pups harboring the R26GRR allele displayed strong tDsRed fluorescence throughout their bodies (Figure 6C). However, when female *Rbfox3*‐iCre::R26GRR mice were crossed with male wild‐type mice, both green and red fluorescent mice were obtained, regardless of the presence of the Cre gene (Figure 6D,E). These results demonstrated that a high incidence of Cre recombination was induced in male germ cells, whereas approximately 50% of female germ cells exhibited Cre recombination activity in *Rbfox3*‐iCre mice.

## 4 DISCUSSION

The Cre‐loxP system is a powerful tool for investigating cell type‐specific gene functions in vivo. As the NeuN antigen/RBFOX3 is a well‐established mature neuron marker, *Rbfox3*‐driven Cre driver mice seem highly useful for developmental biology and neuroscience research. In the present study, we successfully established a novel *Rbfox3*‐iCre knock‐in mouse model and characterized its features, including the effects of iCre knock‐in on endogenous *Rbfox3* expression and functions, as well as the cell‐type specificity of gene recombination.

Although we took advantage of the bicistronic expression system using an IRES element to generation *Rbfox3*‐iCre mice without disruption the *Rbfox3* gene, *Rbfox3* expression was reduced in the heterozygous and homozygous knock‐in mice (Figure 2A,B,D). Previous studies reported that the insertion of IRES‐Cre cassette into the 3′ UTR results in reduction of endogenous gene expression.^22^, ^28^ A somatostatin‐IRES‐Cre mouse model exhibited a massive decrease in somatostatin peptides in the brain, affecting somatostatin‐related neuroendocrine responses in a manner similar to somatostatin‐deficient mice.^28^ Regarding *Rbfox3*‐deficient mice, conventional deletion of *Rbfox3* leads to a reduction in brain weight.^7^ Furthermore, heterozygous and homozygous conditional knockout of *Rbfox3* by *Gad2*‐Cre, a Cre driver for gamma‐aminobutyric acid (GABA) ergic neurons, results in a lower survival rate within 60 d postnatally.^24^ In our mouse model, homozygous *Rbfox3*‐iCre mice showed an approximately 50% reduction in RBFOX3 expression in the brain compared to wild‐type mice, while these mice did not exhibit a reduction in brain weight or an abnormal survival rate (data not shown). In addition, the reduction in neural‐related protein expression and abnormalities in neurogenesis observed in *Rbfox3*‐deficient or ‐conditional knockout mice were not detected in *Rbfox3*‐iCre mice (Figure 2D,E), suggesting that RBFOX3 function is retained in *Rbfox3*‐iCre mice. However, more detailed behavioral analyses are required to precisely assess the influence of IRES‐iCre knock‐in on brain function. Using *Rbfox3*‐iCre mice without the flox allele as a control group may be effective for precisely interpreting the data obtained from conditional knockout mice harboring the *Rbfox3*‐iCre allele.

As expected, iCre activity in the brain was observed in most neurons and was not detected in NeuN (*Rbfox3*)‐negative neural cells, such as Purkinje cells, mitral cells, and other glial cells. On the contrary, in peripheral tissues, *Rbfox3*‐iCre mice exhibited Cre recombination activity in the non‐neural cells. In the bladder, detrusor smooth muscle cells exhibited Cre recombination activity in *Rbfox3*‐iCre mice (Figure 5E). A previous study indicated that detrusor muscles have a transcriptional signature related to both their contractile function of detrusor muscles and neuronal cell type, including the *Rbfox3* gene.^29^ Therefore, recombination activity in the detrusor muscles of *Rbfox3*‐iCre mice may reflect a unique *Rbfox3* expression pattern in the bladder. In cardiac tissue, gene recombination was observed in embryonic *Rbfox3*‐iCre::R26GRR mice (Figure 3), consisting of endogenous *Rbfox3* gene expression in the embryonic heart (Figure S1). The gene recombination in the heart occurred in a mosaic manner (Figure 5C). Considering that the endogenous *Rbfox3* gene is not expressed in the adult heart, our results suggest that the mosaic cardiac gene recombination is established by transient activation of *Rbfox3*‐iCre expression in a subpopulation of cardiomyocytes in the embryonic heart. Germline recombination is a major undesired consequence of site‐specific gene recombination and occurs in over half of the 64 Cre driver lines commonly used for neuroscience research.^30^, ^31^ The reason why germline recombination is broadly observed in various Cre driver models remains unclear. As male *Rbfox3*‐iCre mice exhibit 100% gene recombination in their sperm (Figure 6B), the genotype of their pups could be easily predicted when crossed with flox mice. However, the deletion allele must be detected when female *Rbfox3*‐iCre mice are used to generate conditional knockout mice. Researchers must always be aware of whether the offspring are truly conditional knockout mice, or if one allele is a global knockout. In summary, the *Rbfox3*‐iCre mouse model, capable of inducing characteristic gene recombination in mature neurons as well as in the bladder and heart, holds potential as a novel tool for research in both neuroscience and these peripheral tissues. For example, transcriptional heterogeneity of cardiomyocytes has been observed in both human and model animal hearts and is thought to play a role in the development of heart disease.^32^, ^33^ Alongside AAV‐based mosaic knockout systems,^34^, ^35^ cardiac mosaic gene manipulation using *Rbfox3*‐iCre mice may serve as a valuable new tool for research in cardiac cell biology. However, when conducting gene function analysis using conditional knockout models with *Rbfox3*‐iCre, it is crucial to consider the functional interactions between the brain and the heart, as well as between the brain and urinary muscles.

Collectively, *Rbfox3*‐iCre mice have the potential to recombine genes in neurons of the CNS, while exhibiting a non‐neural recombination signature in peripheral tissues. As one approach to avoiding recombination in peripheral tissues, *Rbfox3*‐iCre mice may be used for neuron‐specific Cre‐dependent gene expression based on a viral gene transduction system, as regional and temporal control is achieved by the stereotaxic injection of viruses. Our study provides a novel tool for developmental biology and neuroscience research and offers characteristic information on employing the *Rbfox3* gene as a driver for external gene expression.

## AUTHOR CONTRIBUTIONS

S. Nishino, M. Hashimoto, S. Mizuno, and K. Murata conceived and designed the study; S. Nishino, M. Hashimoto, S. P. Biswas, N. Mikami, Y. Hasegawa, and K. Murata performed the research and acquired the data; S. Nishino, M. Hashimoto, H. Suzuki, W. Kang, S. Mizuno, and K. Murata analyzed the data. All the authors were involved in drafting and revising the manuscript.

## CONFLICT OF INTEREST STATEMENT

The authors declare that they do not have any competing interests.

## Supporting information


Figures S1–S2.

## Data Availability

The data that support the findings of this study are available in the Materials and Methods, Results, and/or Supplemental Material of this article.
