# Supplementary material for: Characterization of the Rbfox3‐IRES‐iCre knock‐in mouse: Revealing gene recombination activity in neural and non‐neural peripheral tissues
Source: FASEB Bioadv. 2025 Feb 13;7(4):e70003. doi: 10.1096/fba.2024-00143 (PMC11980809; doi:10.1096/fba.2024-00143)
Supplement: Supplementary file 1 — Figures S1–S2. [file FBA2-7-e70003-s001.pdf]

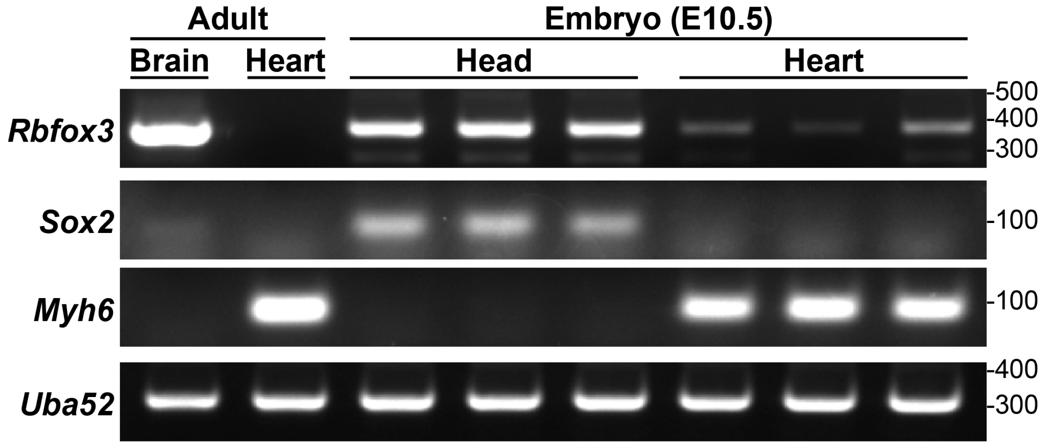

Figure S1. ***Rbfox3*** **is expressed in fetal heart but not in adult heart.**

cDNA prepared from adult brain, adult heart, fetal head, and fetal heart tissues was used for RT-PCR analysis. *Sox2* and *Myh6* were employed as markers for the developing central nervous system and heart, respectively. *Uba52* served as an internal control. The position of the DNA ladder is indicated alongside the images.

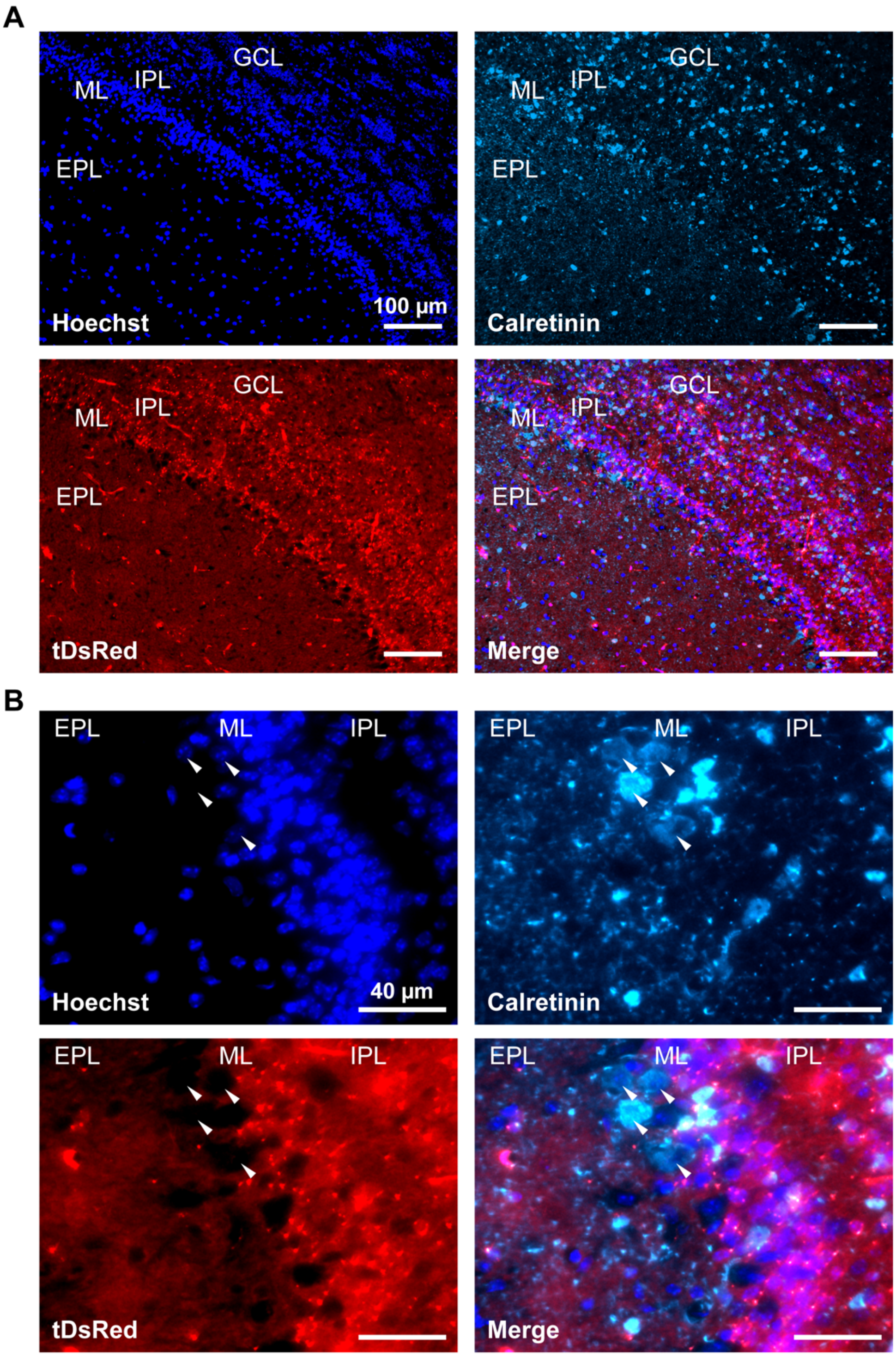

Figure S2. Cre recombination in the olfactory bulb of adult *Rbfox3*-iCre::R26GRR mice.
(A) Low-magnification and (B) high-magnification images of the olfactory bulb stained with an anti-Calretinin antibody and Hoechst, showing the layered structure of the olfactory bulb. The positions of the external plexiform layer (EPL), mitral cell layer (ML), internal plexiform layer (IPL), and granule cell layer (GCL) are indicated in the figures. Mitral cells are marked with arrowheads.
